# Intestinal eosinophils: characterization of elusive granulocytes as anti-bacterial and immunomodulatory effector cells in colitis

**DOI:** 10.1038/s41392-023-01581-7

**Published:** 2023-08-28

**Authors:** Tim Holland, Jochen Mattner

**Affiliations:** 1 Mikrobiologisches Institut - Klinische Mikrobiologie, Immunologie und Hygiene, Universitätsklinikum Erlangen and Friedrich-Alexander-Universität (FAU) Erlangen-Nürnberg, Erlangen, Germany; 2 Medical Immunology Campus Erlangen, FAU Erlangen-Nürnberg, Erlangen, Germany

**Keywords:** Immunological disorders, Antigen processing and presentation

In a recent study published in Nature, Gurtner and colleagues identify active eosinophils (A-Eos) as eosinophil subset with anti-bacterial and immune regulatory functions during intestinal disease.^1^ Due to the combination of human singe cell transciptomics with pre-clinical experimental studies, the authors suggest eosinophils as promising targets for clinical intervention in inflammatory bowel disease (IBD).

Eosinophils are known to exhibit versatile functions at mucosal surfaces. Thus, they can sustain the intestinal epithelial barrier as well as intestinal tissue architecture, communicate with other cell subsets and regulate local immune responses.^2^ However, despite having been associated with a panoply of different diseases including eosinophilic esophagitis or IBD,^3^ their role and plasticity in gastrointestinal disorders have not been well characterized. The present study resolves the transcriptional and functional heterogeneity of eosinophils under steady-state conditions and during gastrointestinal inflammation.^1^

**Fig. 1:**
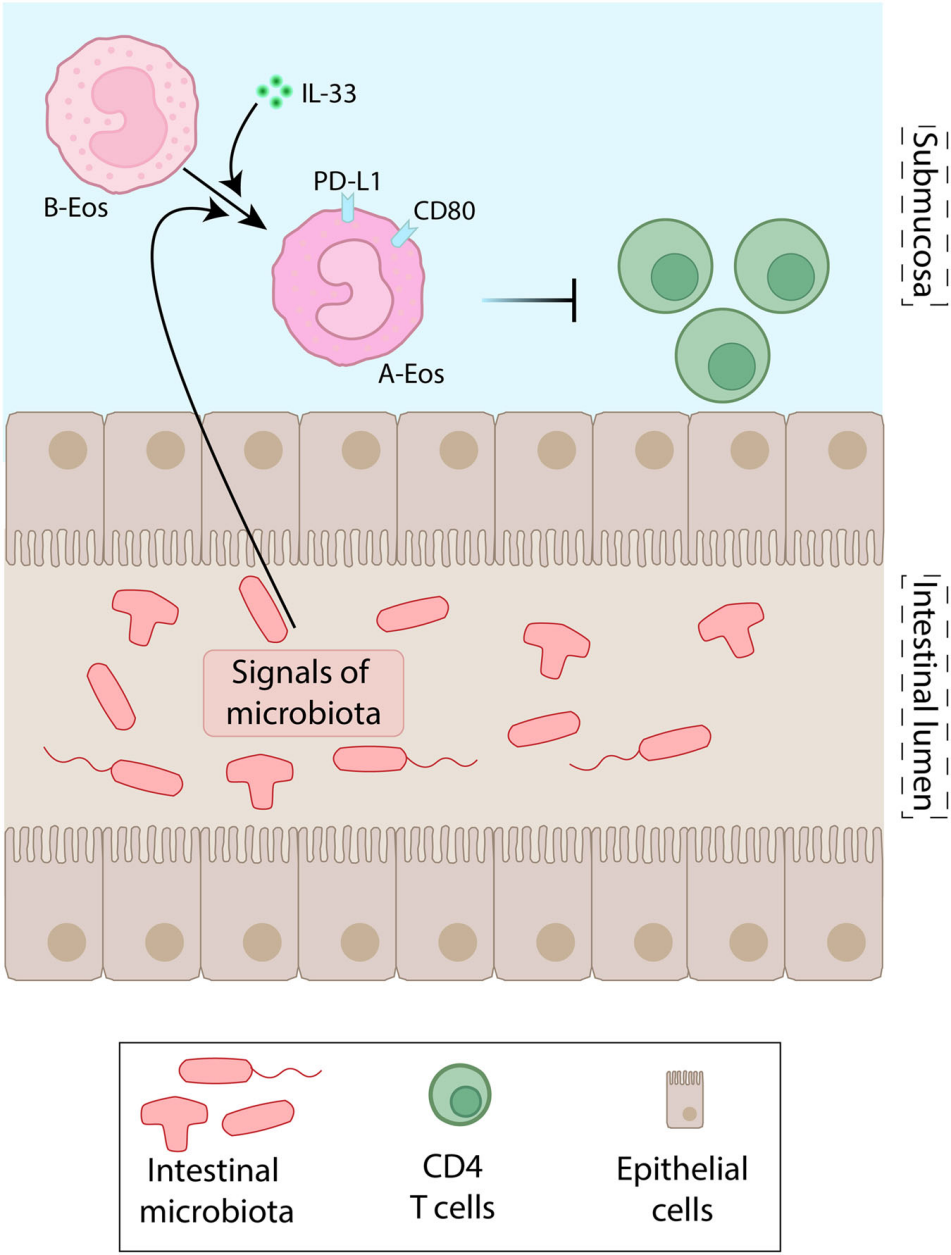
A-Eos protect from intestinal inflammation and infection. Locally produced IL-33 and/or signals derived from intestinal microbiota promote the conversion of eosinophil intermediates (B-Eos) into A-Eos. This transformation is associated with the acquisition of CD80 and PD-L1 expression. A-Eos are anti-bacterial and immunomodulatory effector cells, that limit antigen presentation and restrain the proliferation of colitogenic CD4 T lymphocytes

Single-cell transcriptomes of eosinophils isolated from the stomach, the small intestine, and the colon of IL-5-transgenic mice were compared to colon tissue microarrays obtained from healthy individuals or from IBD patients. Murine eosinophil subsets exhibited distinct transcriptional profiles across tissues and diverged in their cytokine, effector-molecule, and receptor repertoire, indicating that they can exhibit specific functions in individual organs. Defining CD80 and PD-L1 (CD274) as markers in mice and major basic protein (MBP) and PD-L1 as markers in humans, the authors characterize active eosinophils (A-Eos) and basal eosinophils (B-Eos) as two specific functional subsets of the gastrointestinal tract (Fig. 1). A-Eos were detected closer to the intestinal lumen whereas B-Eos were retained nearby the submucosa. Moreover, A-Eos were two to fivefold enriched in tissue samples from the gut of IBD patients. Both intestinal eosinophil subsets differ also functionally and phenotypically from lung-resident populations and from recruited inflammatory eosinophils for which CD101 and CD62L were used as markers of distinction.^4^

To assess the functional role of A-Eos, three experimental models affecting the colon and the stomach were applied. These included acute *Citrobacter* (*C*.) *rodentium* and chronic *Helicobacter pylori* infection as well as acute dextran sulfate sodium (DSS)-induced colitis. Reflecting the situation in IBD patients, intestinal A-Eos expanded in all three pre-clinical models. Using scRNA-seq in combination with in vitro differentiation and in vivo fate mapping analyses, the authors identified B-Eos and circulating eosinophils as transitional differentiation intermediates that can transform into A-Eos. A-Eos exhibited a significantly enhanced bactericidal activity compared to circulating eosinophils, B-Eos, or immature eosinophils. Thus, A-Eos play a crucial role in bacterial clearance and the suppression of colonic immunopathology in *C. rodentium*-infected mice. Across all three gastrointestinal inflammation models, A-Eos specifically upregulated sets of genes that are involved in immune modulation, IFNγ signaling, and MHC-I-restricted antigen processing and presentation. In vitro co-culture studies revealed an enhanced CD8 T cell proliferation in an antigen-dependent manner while only purified intestinal A-Eos, and not B-Eos, restrained the proliferation of CD4 T cells (Fig. 1). Accordingly, eosinophil-deficient mice developed more severe DSS-induced colitis. Thus, the acquisition of anti-bacterial and immunomodulatory functions accompanies the conversion of B-Eos and circulating eosinohils into A-Eos.

IL-5 is recognized as a major maturation, survival, and differentiation factor of eosinophils.^5^ Of note, Gurtner and colleagues did not detect any transgene-specific effects between A-Eos and B-Eos from B6 and IL-5 - transgenic mice. A-Eos and B-Eos similarly required eotaxin–CCR3 interactions for the accumulation in gastrointestinal tissues. Furthermore, anti-IL-5 treatment affected both eosinophil subsets in a comparable manner.

Single-cell regulatory network inference and clustering (SCENIC) combined with in vitro genome-wide CRISPR inhibition screens identified locally produced IL-33 as an essential and specific cytokine for the maturation and differentiation of intestinal A-Eos (Fig. 1). A-Eos expressed the IL-33 receptor (IL-33R, ST2) at higher levels compared to B-Eos. Moreover, the IL-33R was not detected in eosinophils from other organs, including the lung, adipose tissues, uterus, peritoneum, or thymus. Accordingly, IL33-deficient mice exhibited reduced A-Eos frequencies at steady state in the small intestine and the stomach, but not in the colon. The neutralization of the IL-33-ST2-MyD88 axis in vitro hampered also the differentiation of conditioned bone marrow-derived eosinophils into A-Eos, as indicated by a lack of CD80 and PD-L1 induction. As frequencies of colonic A-Eos were significantly reduced in germ-free mice as well as in SPF mice following depletion of commensal gut microbiota by broad-spectrum antibiotics, the authors suggested that alternative, microbiota-dependent signals might underlie the differentiation of A-Eos in the colon as well (Fig. 1). Interestingly, an application of recombinant IL-33 in vivo increased the numbers of A-Eos in the colon in a MyD88-dependent manner.

SCENIC analyses revealed also an increased signaling downstream of IFNγ in A-Eos during inflammation. An application of IL-33 induced NF-κB signaling and the expression of markers specific for A-Eos, whereas IFNγ treatment upregulated the expression of CD274 and genes involved in antigen presentation. Simultaneous addition of IL-33 and IFNγ endowed bone marrow-derived eosinophils to restrain the proliferation of CD4 T cells and to downregulate the expression of granular protein and anti-microbial genes. An application of IFNγ in vivo potentiated also the effects of IL-33 under steady-state conditions and increased colonic A-Eos numbers to levels observed during colitis.

Similar to in colitic tissues of mice, CD4 T cell transcripts co-localized with SIGLEC8 transcripts, a selective marker for eosinophils in humans, in colonic sections of IBD patients as indicated by in situ RNA imaging. SIGLEC8 RNA molecules in close proximity to CD4 molecules associated with signaling components of NF-κB and IFNγ signaling pathways. Thus, the same pathways presumably drive interactions between A-Eos and CD4 T cells in mouse and human colitis.

In summary, the role of intestinal eosinophils in the pathogenesis of IBD remains enigmatic. Moreover, conflicting results have been published. In this context, distinct experimental settings or differences in the community composition of intestinal microbiota can influence the phenotype of individual cell subsets and thus, the outcome of a variety of murine models used in biomedical research. In the present study, Gurtner and colleagues introduce A-Eos as anti-bacterial and immunomodulatory effector cells.^1^ Thus, in acute experimental colitis A-Eos and IL-33 which perpetuates the conversion of B-Eos into A-Eos exhibit anti-colitogenic effects. Vice versa, however, IL-33 has been also reported to promote experimental colitis. Thus, IL-33 might contribute to the differentiation of Th9 cells that trigger colitogenic responses, particularly associated with ulcerative colitis.^6^ Moreover, eosinophils themselves might exacerbate chronic colitis in distinct experimental settings.^5^ Thus, targets of eosinophil conversion must be carefully and thoroughly assessed before being addressed in IBD patients. Whether eosinophils are suitable targets for the treatment of IBD is therefore subject to further investigation.
